# Transfer of skills and comparison of performance between king vision® video laryngoscope and macintosh blade following an AHA airway management course

**DOI:** 10.1186/s12871-016-0296-9

**Published:** 2017-01-10

**Authors:** Lukas E. Wolf, José A. Aguirre, Christian Vogt, Christian Keller, Alain Borgeat, Heinz R. Bruppacher

**Affiliations:** 1 Institute of Anesthesiology, University Hospital Zurich, Zurich, Switzerland; 2 Department of Anesthesiology, Schulthess Clinic, Schulthess Clinic, Lengghalde 2, 8008 Zurich, Switzerland; 3 Division of Anesthesia, Balgrist University Hospital, Zurich, Switzerland; 4 SkillsLab, Deanery, Faculty of Medicine, University of Zurich, Zurich, Switzerland

**Keywords:** Airway management, Medical education, Endotracheal intubation, Video laryngoscopy, Resuscitation, Advanced life support

## Abstract

**Background:**

To potentially optimize intubation skill teaching in an American Heart Association® Airway Management Course® for novices, we investigated the transfer of skills from video laryngoscopy to direct laryngoscopy and vice versa using King Vision® and Macintosh blade laryngoscopes respectively.

**Methods:**

Ninety volunteers (medical students, residents and staff physicians) without prior intubation experience were randomized into three groups to receive intubation training with either King Vision® or Macintosh blade or both. Afterwards they attempted intubation on two human cadavers with both tools. The primary outcome was skill transfer from video laryngoscopy to direct laryngoscopy assessed by first attempt success rates within 60 s. Secondary outcomes were skill transfer in the opposite direction, the efficacy of teaching both tools, and the success rates and esophageal intubation rates of Macintosh blade versus King Vision®.

**Results:**

Performance with the Macintosh blade was identical following training with either Macintosh blade or King Vision® (unadjusted odds ratio [OR] 1.09, 95% confidence interval [95% CI] 0.5–2.6). Performance with the King Vision® was significantly better in the group that was trained on it (OR 2.7, 95% CI 1.2–5.9). Success rate within 60 s with Macintosh blade was 48% compared to 52% with King Vision® (OR 0.85, 95% CI 0.4–2.0). Rate of esophageal intubations with Macintosh blade was significantly higher (17% versus 4%, OR 5.0, 95% CI 1.1–23).

**Conclusions:**

We found better skill transfer from King Vision® to Macintosh blade than vice versa and fewer esophageal intubations with video laryngoscopy. For global skill improvement in an airway management course for novices, teaching only video laryngoscopy may be sufficient. However, success rates were low for both devices.

**Electronic supplementary material:**

The online version of this article (doi:10.1186/s12871-016-0296-9) contains supplementary material, which is available to authorized users.

## Background

Video laryngoscopy (VL) is promoted as rescue technique in case of failed direct laryngoscopy (DL) [1, 2], or as the primary approach to intubation, especially in emergency medical systems or in intensive care where proficiency with DL may be limited [3–5], first attempt success is important for patient safety [6] and unexpected difficult intubations are frequent [7].

A comparative study in the operating theater between VL with GlideScope® (Verathon Medical, Burnaby BC, Canada) and DL showed very high success rate of 93% with VL versus 51% with DL when performed by health professionals inexperienced in laryngoscopy [8]. However, VL equipment is not always available and the presence of fluids, sunlight or camera fogging may increase difficulty with VL [9]. Therefore direct laryngoscopy skills may still be required for health care providers attempting endotracheal intubation.

A variety of video laryngoscopes have been introduced to clinical practice with different blade designs and handling requirements [2]. A recent study by Kleine-Brueggeney et al. compared the performance of six different systems in a simulated difficult airway (cervical collar) and found clinically relevant differences in success rates as well as rate of tissue trauma, highlighting the importance of blade design [10]. For our study we chose the King Vision® (KV) video laryngoscope (King Systems®, Noblesville, Indiana, USA), which is available with a standard or channeled blade. Akihisa et al. showed better performance with the channeled version for inexperienced operators [11] and therefore we used the channeled blade for this trial. Used with the channeled blade, the KV belongs to the family of “channeled rigid indirect optical devices”, like the Airtraq® (Prodol Meditec SA, Vizcaya, Spain) or Pentax-Airway Scope® (Hoya, Tokyo, Japan) [2].

Many courses such as the American Heart Association® (AHA) Advanced Cardiac Life Support® (ACLS) or the AHA Airway Management Course® (AMC) include training of intubation skills on a manikin. These courses target airway management providers with limited skills and it is unclear whether DL, VL or both should be taught. While evidence suggests skill transfer from DL to VL is limited [12–14] yet current practice in the AMC is to teach DL only. Our hypothesis is that changing current practice to teaching VL in such courses might lead to better overall skill gain. Therefore our primary outcome was skill transfer from VL to DL as assessed by first attempt success rates within 60 s with the Macintosh blade (MAC) after having trained DL or VL only. Secondary outcomes were skill transfer from DL to VL, the efficacy of teaching both tools, the success and esophageal intubation rates of DL versus VL and the learning effect of the AMC.

## Methods

### Participants and study groups

After receiving an IRB waiver, 90 volunteers (medical students, residents and staff physicians) were recruited for participation in an AMC free of charge in April and June 2014.

We randomized participants into one of three intervention groups: One group trained DL with MAC only (DL-group), one trained VL with the KV only (VL-group) and one group trained both DL and VL (DL + VL-group). Randomization was performed block wise for physicians and students. A fourth control group of 22 volunteers was recruited to participate only in the testing sessions without prior training (NT-group) to evaluate the learning effect of the AMC. Of these 112 participants we lost 8 who attended the AMC but could not be recruited for the testing session afterwards (DL-group 3, VL-group 3, DL + VL-group 2). Written informed consent was obtained from all participants.

Demographic data included: gender; field of clinical practice (operative versus non-operative); number of prior attempted or observed intubations; total number, and number within the past three months, of simulation-based training courses that included intubation training on manikins; years of experience (sum of years in medical school, years of post-graduate training and clinical practice as a physician).

**Fig. 1:**
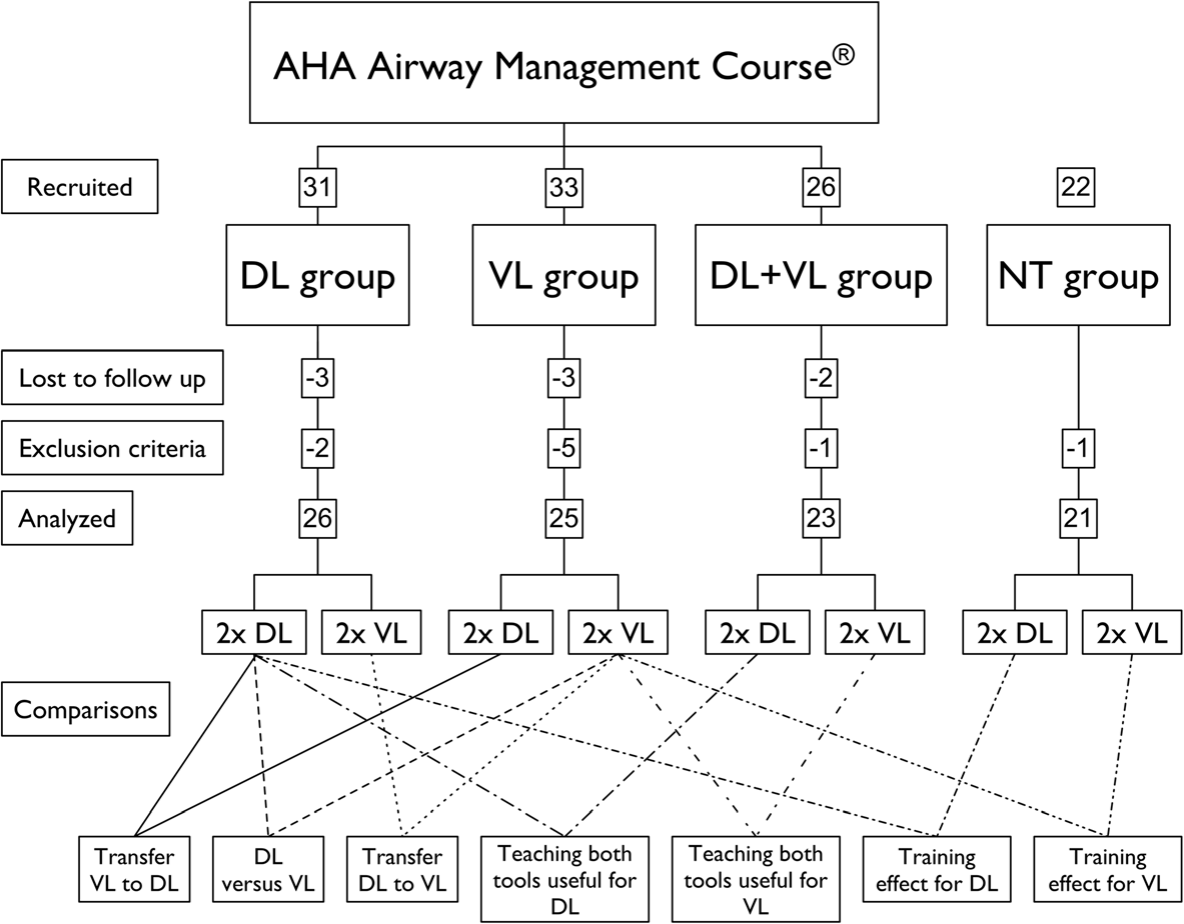
Study design – flow of participants and analysis. *Full line*: primary outcome; *dashed lines*: secondary outcomes. DL: direct laryngoscopy; VL: video laryngoscopy; NT-group: not trained group

Exclusion criteria were previous experience in endotracheal intubation in humans, having visited advanced life support or airway management courses within the past 3 months or having seen or performed intubations between the AMC and the testing session. Figure 1 illustrates the flow of participants and the study design.

### Airway management course

Our AMC strictly followed the AMC instructor guidelines. Four trained anesthesiologists served as course instructors. All groups received the obligatory part of the AMC (basic airway management) and additional sections with the laryngeal mask airway and endotracheal intubation. Total time for the course was 3.5 h. As per course guidelines, the intubation part included the official course videos (20 min) and hands on training for approximately 50 min, including a test. Target group size was 6 (actual range 2–8). Airway training was performed on Laerdal Airway Management Trainers® (Laerdal, Stavanger, Norway). The courses were held at Balgrist University Hospital, University Hospital Zurich and the University of Zurich.

### Testing protocol

Following the AMC, within 6 weeks, participants were required to attempt intubation on two (out of four) human cadavers at the Institute of Anatomy, University of Zurich. For this trial, Thiel-embalmed cadavers were used to ensure optimal fidelity with respect to mechanical tissue elasticity and anatomy [15]. Because intubation attempts may lead to tissue damage we used two sets of two cadavers for the tests in alternating order. Intubation of each cadaver with MAC size 4 was found to be easy by at least four experienced anesthesiologists (partial or complete view of the vocal cords with median time to intubation of 12 s, range 5 to 45 s).

Each participant received a short verbal instruction for both devices. For the NT-group we expanded this instruction to two minutes. Participants had one attempt with a MAC size 4 and the KV in random order on both cadavers, resulting in a total of 4 intubation attempts per participant. During the intubation attempts no instructions were given, but participants were stopped if they were deemed to be in danger of damaging dental or oral tissues of the cadaver. The supervising anesthesiologist checked tube placement via laryngoscopic view. All intubations were videotaped (participants hands and forearms only) and times noted by two raters blinded to the study group. The time was taken from insertion of the blade into the mouth until tube placement.

### Primary outcome

For our primary outcome, transfer of skills from VL to DL, we compared success rates within 60 s with MAC (direct laryngoscopy) between the DL- and VL-group. Failure to reject the null hypothesis (equal performance even when not trained with DL) would signify at least partial transfer of skills from VL to DL.

### Secondary outcomes

To assess the transfer of skills from DL to VL we compared success rates within 60 s with the KV between the VL- and DL-group.

To evaluate the benefit of teaching both tools for video laryngoscopy, we compared success rates with KV between the VL- and DL + VL-group. Similarly, we compared the success in direct laryngoscopy with MAC between the DL- and DL + VL-group.

The performance of MAC versus KV was evaluated by comparing the success rates within 60 s between the DL-group with MAC and the VL-group with KV. Additionally, we compared the occurrence of esophageal intubations between these two groups.

To evaluate the learning effect of the AMC for DL, we compared performance between the NT- and DL-group. Similarly for VL, we compared performance between the NT- and VL-group. Table 1 gives an overview of all comparisons performed to answer our study questions.

**Table 1:** Data analysis plan - overview of comparisons

Question	Comparison between groups
Transfer of skill from VL to DL?	DL-group and VL-group with MAC
Transfer of skill from DL to VL?	VL-group and DL-group with KV
Teaching both tools beneficial for VL?	VL-group and DL + VL-group with KV
Teaching both tools beneficial for DL?	DL-group and DL + VL-group with MAC
MAC versus KV?	DL-group with MAC and VL-group with KV
AMC successful for VL?	VL-group and NT-group with KV
AMC successful for DL?	DL-group and NT-group with MAC

*DL*, direct laryngoscopy, *MAC* Macintosh blade, *KV* King Vision®, *NT-group* not trained group, *VL* video laryngoscopy

### Statistical analysis

Data analysis was performed with the open-source R Statistics Package (Version 3.2.1). Categorical and continuous demographic data was analyzed with the *χ* ^2^-Test, Fisher’s exact test or Kruskal-Wallis rank sum test, as appropriate. *P* values below 0.05 were considered significant. Intubation success within 60 s was analyzed by binary logistic regression with cluster-robust standard errors to account for multiple dependent measurements per participant (two intubations with each device on different cadavers). We constructed a logistic regression model with the following variables: operative profession, cadavers tested on and physician versus medical students. Other variables tested for inclusion into this model but found to be non-significant were gender, experience level, the instructor leading the AMC, number of observed intubations and earlier courses with intubation training. This model was used to calculate adjusted odds ratios. Results are reported as unadjusted and adjusted odds ratios (OR) with 95% confidence interval (95% CI). Occurrence of esophageal intubation was analyzed accordingly. 95% CI for success rates within 30 and 60 s were calculated from cluster-robust standard errors.

## Results

**Fig. 2:**
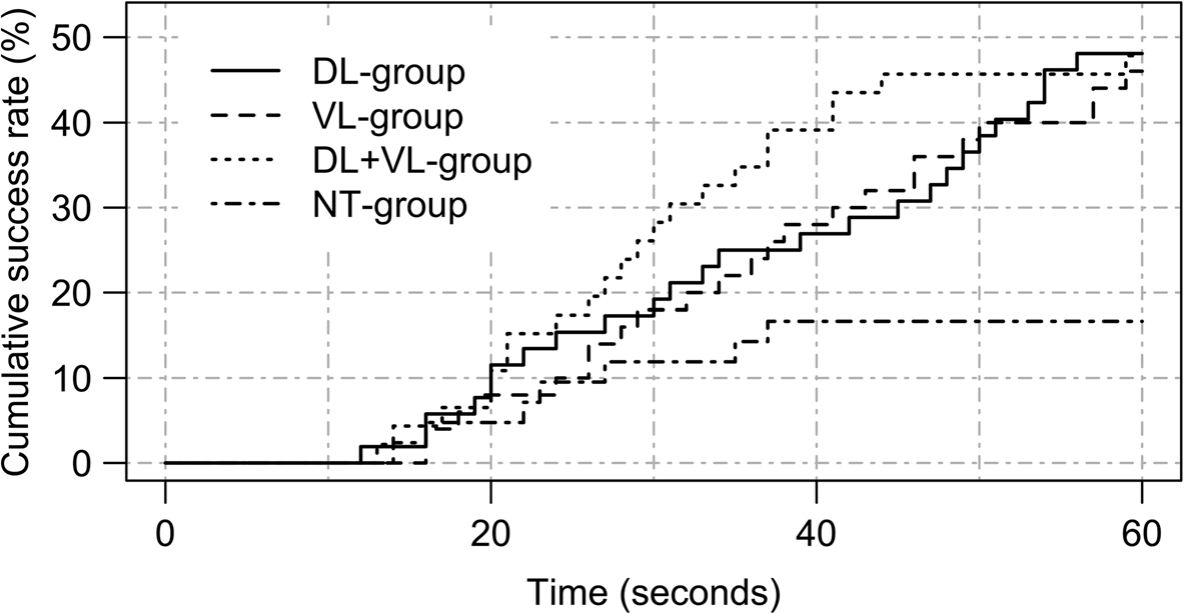
Time to achieve successful intubation with the Macintosh blade by study group (time to event plot). DL, direct laryngoscopy; NT-group, not trained group; VL, video laryngoscopy

**Fig. 3:**
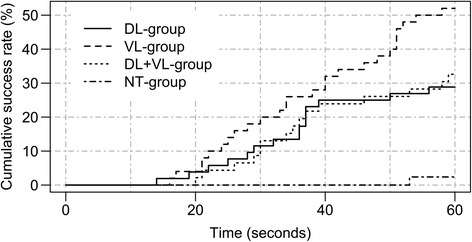
Time to achieve successful intubation with the King Vision® by study group (time to event plot). DL, direct laryngoscopy; NT-group, not trained group; VL, video laryngoscopy

A total of 104 participants were tested. According to exclusion criteria, 9 participants were excluded from analysis (DL-group 2, VL-group 5, DL + VL-group 1, NT-group 1). Data of 95 participants and 380 intubation attempts was analyzed. Demographic characteristics of the participants are summarized in Table 2. Baseline characteristics between the four groups were similar, but there were more students in the DL + VL-group (and therefore less years of experience). The two cadaver sets were equally distributed between DL-, VL- and DL + VL-group (*p* value 0.93). Percentages of successful and esophageal intubations within 60 s for each group are reported in Table 3. Success rates within 30 s are shown in Table 4. Intubation times with MAC for each group are shown in Fig. 2, with KV in Fig. 3. Raw study data with annotations is provided in the Additional file 1.

**Table 2:** Demography by training groups

Variable/group	DL (*n* = 26)	VL (*n* = 25)	DL + VL (*n* = 23)	NT (*n* = 21)	*P* value^a^
Sex (m/f)	14/12	17/8	11/12	11/10	0.52
Physicians/students	12/14	12/13	7/16	9/12	0.61
Operative profession, n (%)	4 (15)	3 (12)	3 (13)	4 (19)	0.94
Number of earlier courses^b^	0 (0–1)	0 (0–2)	0 (0–1)	0 (0–1)	0.62
Years of experience^b^	6 (4–10)	6 (3–11)	3 (3–7.5)	4 (2–9)	0.25
Prior observed intubations^b^	0 (0–2)	0 (0–3)	0 (0–2)	0 (0–1)	0.43

*DL* direct laryngoscopy, *NT-group* not trained group, *VL* video laryngoscopy

^a^ *P* value was calculated with Fisher’s exact- or *χ* ^2^-test for categorical data and Kruskal-Wallis test for continuous data

^b^Result as median (1st and 3rd quartile)

**Table 3:** Successful and oesophageal intubations within 60 s with Macintosh blade (MAC) and King Vision® (KV)

Study group	Success (%)		Oesophageal intubation (%)	
	KV	MAC	KV	MAC
DL-group (*n* = 52)	29 (18–42)^a^	48 (32–64)^c^	2 (0–12)	17 (10–28)^b^
VL-group (*n* = 50)	52 (39–65)^a, c, d^	46 (32–61)	4 (1–14)^b^	14 (6–28)
DL + VL-group (*n* = 46)	33 (24–43)^d^	48 (33–63)	2 (0–14)	17 (10–29)
NT-group (*n* = 42)	2 (0–15)^c^	17 (8–32)^c^	0	24 (14–38)

Results in % (95% CI) of number of attempts (n). *DL* direct laryngoscopy, *NT-group* not trained group; *VL*, video laryngoscopy

^a^Significant difference (unadjusted OR 2.7, 95% CI 1.2–5.9) for skill transfer from DL to VL

^b^Significant difference (unadjusted OR 5.0, 95% CI 1.1–23.1)

^c^Significant difference (OR and 95% CI in text) for course learning effect

^d^Significant difference (unadjusted OR 2.2, 95% CI 1.1–4.4) for teaching both tools

**Table 4:** Successful intubations within 30 s

Study group	First attempt success rate (%)	
	King Vision®	Macintosh blade
DL-group (*n* = 52)	12 (5–25)	19 (9–36)
VL-group (*n* = 50)	20 (10–35)	18 (10–29)
DL + VL-group (*n* = 46)	13 (6–25)	28 (17–43)
NT-group (*n* = 42)	0	12 (5–24)

Results in % (95% CI) of number of attempts (n). *DL* direct laryngoscopy, *NT-group* not trained group, *VL* video laryngoscopy

### Primary outcome: transfer of intubation skills from KV to MAC

Comparing success rates with MAC between the DL- and VL-group, we found a non-significant unadjusted OR of 1.09 (95% CI 0.5–2.6) and adjusted OR of 1.07 (95% CI 0.4–2.6). Thus, performance with MAC was the same if trained with KV only, indicating at least partial transfer of skills from KV to MAC.

### Secondary outcomes

#### Transfer of intubation skills from MAC to KV

Comparing success rates with the KV between the VL- and DL-group we found a significant unadjusted OR of 2.7 (95% CI 1.2–5.9) and adjusted OR of 3.0 (95% CI 1.4–6.6). Thus, there was a significantly higher success rate with the KV in the group trained with the device, indicating an incomplete transfer of skills from MAC to KV.

### Benefit of teaching both tools

Comparing performance with MAC between the DL- and DL + VL-group we found a non-significant unadjusted OR of 1.01 (95% CI 0.4–2.5) and adjusted OR of 1.00 (95% CI 0.4–2.7). Inversely for performance with KV between the VL- and DL + VL-group we found a significant unadjusted OR of 2.2 (95% CI 1.1–4.4) and adjusted OR of 2.4 (95% CI 1.3–4.8). Therefore in this setting, there was no benefit for being trained with both tools for DL and performance with VL was significantly worse in the DL + VL group.

### DL versus VL

The success rate within 60 s for the DL-group with MAC was 48% compared to 52% for the VL-group with KV. This resulted in a non-significant unadjusted OR of 0.85 (95% CI 0.4–2.0) and adjusted OR of 0.78 (95% CI 0.3–1.8). With MAC, the rate of esophageal intubations within 60 s was 17% compared to only 4% with KV resulting in a significant unadjusted OR of 5.0 (95% CI 1.1–23) and adjusted OR of 5.9 (95% CI 1.4–25).

### Learning effect of the AMC

Comparison of success rates within 60 s for MAC between the DL- and NT-group showed a significant unadjusted OR of 4.6 (95% CI 1.6–14) and adjusted OR of 3.4 (95% CI 1.0–12). Accordingly for KV we found a significant unadjusted OR of 44 (95% CI 5.8–337) and adjusted OR of 48 (95% CI 7.2–320) between the VL- and NT-group.

## Discussion

We analyzed intubation performance of novice airway management providers after training with either KV, MAC or both: In our primary outcome, transfer of skill from VL to DL, we found no significant difference, indicating at least partial transfer of skills from KV to MAC. In our secondary outcomes we found worse performance with KV for the DL-group compared to the VL-group, indicating incomplete transfer of skills from DL to VL. Training both tools did not increase performance for DL and resulted in worse performance for VL, but there are several possible biases for this result as discussed below. Success rates for both MAC and KV were similar but esophageal intubation was much less common with VL. Lastly, our AMC did increase performance of participants when compared to no training at all.

### Transfer of skills

Performance with DL was not dependent on the study group, indicating that among those inexperienced in airway management, there is at least partial transfer of the skills acquired in training with the KV to DL with MAC. The VL-group on the other hand performed significantly better with the KV than the DL-group which was not trained with the KV. Therefore, transfer of skills from DL to VL is incomplete for our novice participants. Accordingly, studies examining providers who are experienced in DL found worse performance with VL when experience with VL was lacking [12, 14]. Burnett et al. [3] and Jarvis et al. [4] observed the need for a different technique when intubating with KV with the channeled blade. Often intubation was unsuccessful despite good visualization of the vocal cord. We, in turn, observed that intubation failed when the KV was inserted too deeply or not in the midline so that the guided endotracheal tube ended up too far posterior or deviated laterally. Withdrawing the KV seems to be the solution in these cases. Our observation is supported by data from Gu et al., who showed better results with the GlideScope® when the laryngoscopic view was deliberately restricted to only partial view of the vocal cords [16]. In their study on learning DL, Mulcaster et al. found proper insertion and lifting of the laryngoscope blade to be the most critical part for intubation success [17]. To find the optimal insertion depth and for proper manipulation of the blade, a thorough understanding of airway anatomy is needed which seems being acquired with VL training.

### Benefit of teaching both tools

With MAC the DL + VL-group performance was similar to the DL-group. Having trained VL as well as DL does not seem to offer an additional benefit. Regarding performance with the KV, we saw a significantly worse success rate in the DL + VL-group compared to the VL-group. The DL + VL-group actually performed similar to the DL-group, which received no training with KV. We assume this is a bias because of the focus of the AMC on DL while VL is not discussed or shown in the teaching videos. Therefore participants of the DL + VL-group probably focused on DL while neglecting VL. Alternatively, teaching both tools within the intubation part of the AMC may have been cognitive overload [18]. The DL + VL group included proportionally more students than the other groups (without significance), which might be an alternative explanation for the worse performance. But adjustment for this factor did not change our result and the DL + VL group showed no worse performance with MAC. Therefore our data suggests, that overall skill gain might be best if only VL is taught in the AMC in light of the limited time allocated to hands on training. However, evidence is scarce and in a setting with more time, teaching both tools might be better. Further research would be needed with special considerations given to minimizing the bias introduced by the course structure.

### Comparison of performance between MAC and KV

We found no difference in success rates between MAC and KV. In their review, Niforopoulou et al. state, that in a situation with an easy laryngoscopic view, success rates were equal between DL and VL but VL intubations were slower [19]. As we had good laryngoscopic views in all four cadavers our results seem to be in accordance with previous studies, although our chosen time limit of 60 s may have favoured DL. This contrasts with the results of Nouruzi-Sedeh et al., who found much better performance with GlideScope® compared to MAC in their study of novice airway management providers [8]. As with our cadavers, their patients were selected for easy laryngoscopy and were excluded if an experienced anesthesiologist could not see at least part of the glottic opening. They allowed up to 120 s for the intubation attempt, but DL was even slower than VL. So our cutoff of 60 s would have favored VL even more. Additionally, they used a specialized drill for intubation training (without mentioning the time allocated to hands on training) while we used the limited and standardized time frame mandated by the AMC structure. No comparative study between GlideScope® and KV for inexperienced providers is known to us but for experienced providers first-attempt success rates (85 and 87%) and intubation time (60 and 59 s) were identical [10].

Regarding esophageal intubations, we found a statistically significant lower rate of 4% with KV versus 17% with MAC. This is consistent with previous studies with novice participants [13], with paramedics inexperienced in VL [20] and a recent meta-analysis [21]. This difference may be clinically significant and VL might improve patient safety because of the consequences of unrecognized esophageal intubation.

### Learning effect of the AMC

Participants had very low first attempt success rates of 20% (VL-group with KV) and 19% (DL-group with MAC) within 30 s, the recommended time for intubation attempts according to the AMC course videos (Table 4). The European Resuscitation council and the AHA CPR Guidelines state that chest compressions should be stopped for no more than 10 s while passing the endotracheal tube through the vocal cords, but no maximum time per intubation attempt is given [22, 23]. We chose a cutoff time of 60 s, which is clinically reasonable for intubation attempts when chest compression is not interrupted or in the setting of respiratory compromise with pulse. First attempt success rate within 60 s with KV was 2% in the untrained group compared to 52% for the VL-group. Similarly for MAC, success rate was 17% without training compared to 48% for the DL-group. This demonstrates the benefit of AMC training with rates similar to earlier studies with participants who had no relevant prior experience (39, 42%) [24, 25].

### Limitations

While Thiel-embalmed cadavers are much more lifelike than traditional formalin embalmed cadavers [15], we still cannot exclude a bias from the anatomical properties and the limited number of cadavers.

Some aspects of our courses and testing sessions, like AMC instructor or cadavers, could not be randomized because of organizational limitations. We included these factors in our regression model to adjust for possible bias. Additionally our NT-group was not randomized together with the three intervention groups but was a control group recruited from the same population. However, the NT-group was analyzed only as baseline for assessing the learning effect of the AMC.

Our sample size resulted in relatively broad CI for success rates (Tables 3 and 4). Therefore a clinically relevant difference may go undetected due to limited power. To elaborate, while our results demonstrate better transfer from KV to MAC than vice versa, we cannot quantify this transfer conclusively.

Because of our study design with several secondary research questions, we performed multiple statistical testing which increases the risk of type 1 error.

Generalization of our conclusions to video laryngoscopes of the same class like Airtraq® and Pentax-Airway Scope® seems reasonable. This may be different for video laryngoscopes with Macintosh shaped blades. At least for airway management providers experienced with DL and inexperienced with VL, Alvis et al. found worse performance with the channeled KV compared to the McGrath MAC® [26]. This is expected, as handling of the angulated and channeled KV is different compared to the MAC (as explained above) while the McGrath MAC® usage is similar to a MAC. Therefore we would expect similar or even better transfer of skills from Macintosh shaped VL to DL. Future research is needed to answer whether transfer from DL to VL may be better with other video laryngoscopes than the KV.

Our chosen study setting does not allow prediction of participant’s future learning curve and whether there is a ceiling effect on skill transfer.

## Conclusions

Intubation training in our AMC did increase performance in intubating human cadavers. But as explicitly stated in the AMC course videos and according to ERC and AHA CPR Guidelines 2010, novice participants should be discouraged from attempting endotracheal intubation [22, 23]. This is also valid with the King Vision® video laryngoscope as demonstrated by our data. Instead, bag-mask ventilation or supraglottic airway devices should be used [22, 23].

As for our aim of optimizing intubation skill teaching for novice participants in an Airway Management Course, we found better transfer of intubation skills from video laryngoscopy (with a King Vision® device) to direct laryngoscopy (with a Macintosh Blade) than vice versa and a significant lower rate of esophageal intubations with video laryngoscopy. As unrecognized esophageal intubation is a threat to patient safety, this very low rate may be an important argument for video laryngoscopy. Our findings thus suggest that where there is limited time allocated to intubation training, teaching only video laryngoscopy (with a King Vision® device) to novice participants of an airway management course is possible without compromising advancement in direct laryngoscopy skills. If training time permits in a different course setting, teaching both tools may lead to improved skill acquisition. Future research may focus on this question, as well as on the question of applicability of the results to other video laryngoscopes.

## Additional file

Additional file 1: Raw study data. (XLSX 34 kb)

## Abbreviations

95% CI: 95% confidence interval
AHA: American Heart Association®
AMC: Airway Management Course®
DL: Direct laryngoscopy
KV: King Vision®
MAC: Macintosh blade
NT-group: Not trained group
OR: Odds ratio
VL: Video laryngoscopy
